# Study of Psychomotor Development and Environmental Quality at Shelter Homes for Children Aged 0 to 2 in the Department of Chuquisaca (Bolivia)

**DOI:** 10.3390/ijerph17124191

**Published:** 2020-06-12

**Authors:** Sagrario Pérez-De La Cruz, Ivonne Ramírez, Carolina Maldonado

**Affiliations:** 1 Department of Nursing, Physiotherapy and Medicine, University of Almería, 04120 Almería, Spain; 2 Department of Health, University of San Francisco Xavier de Chuquisaca, 580 Sucre, Bolivia; ramirez.ivonne@usfx.bo; 3 Neurodevelopment Center “Rapha”, University of San Francisco Xavier de Chuquisaca, 580 Sucre, Bolivia; carolina_maldonado@outlook.com

**Keywords:** *casas cuna*, stimulation, psychomotricity therapy, development

## Abstract

Children in situations of destitution who become institutionalized commonly display developmental disorders, including delayed growth. The aim was to evaluate the environmental quality of the *casas cuna* of the Department of Chuquisaca (Plurinational state of Bolivia) in children aged 0 to 2 years old after receiving an early stimulation program based on psychomotor therapy. Thirty-six children who were institutionalized at shelter homes in the Department of Chuquisaca were selected to receive sessions of psychomotricity over a five-month period. The Infant/Toddler Home Observation for Measurement of the Environment (IT-HOME) scale and the Attachment During Stress Scale (ADS) were used. The adult−child relationship with factors of responsiveness (−0.89; *p* = 0.037), acceptance (0.57; *p* = 0.024), organization (−1.03; *p* < 0.001), learning material (−2.57; *p* < 0.001) and involvement (−1.92; *p* < 0.001) scored below expectations, showing that environmental indicators are a poor stimulation for children growing up in shelter homes. Improvements were found in the children’s development after receiving this therapy. In conclusion, an early stimulation program based on psychomotor therapy over five months provided favorable results for the acquisition of skills for communication, motor development and social skills, which positively affect the psychomotor development.

## 1. Introduction

A child’s surrounding environment has a major impact on their life as this marks the first childhood experiences, thereafter influencing their response to both personal and external needs, ranging from their relationship with adults/parents/caregivers to social demands that start with secularization and future adult life [1]. The Convention on the Rights of Persons with Disabilities states that countries should ensure that people with disabilities have access to appropriate health services, including general health care, habilitation and rehabilitation services, and are not discriminated against in the provision of health services [1], as actively supported by the World Health Organization (WHO).

Measuring the quality of the environment among children living in shelter homes and therefore at psychosocial risk is important with regard to both the child’s physical and mental health [2,3]. Childhood attachment is an emotional closeness that allows the child to develop a sense of security and protection towards the world and is currently considered to be a vital condition in ensuring healthy child development [4]. Three styles of attachment are distinguished: 1) safe, healthy attachment; and unsafe attachment styles: 2) anxious attachment and 3) avoidant attachment [5].

Recent research has shown that positive interaction between a child and his or her parents (or other primary caregivers) has a significant impact on the development of the child’s brain [6]. Thus, children seek interaction with adults between birth and three years of age [7]. Furthermore, other studies [3,8] have shown that affective reciprocal relations among institutionalized children are especially critical and dependent on the role fulfilled by the adults within the institution.

Previous studies based on children in situations of destitution and, thereafter, in institutional care [5,7,8] primarily concentrated on determining the children’s development based on different development scales [9,10]. These scales served to verify the impact of the same in situations of social risk and the effect this could have on a child’s growth rate [11] and, concurrently, the favorable effect that early stimulation nurture and the appropriate environment could have on a child’s development [2,3,8,12,13,14,15,16,17,18]. Psychomotricity is, therefore, a discipline that, based on an integral conception of the human being, deals with enhancing the correct interaction that is established between knowledge, emotion, body and movement and its importance to children’s development as well as their capacity to express themselves and interact in a social context [12].

The quality of care and services at nursery schools in countries within the Andes region of South America have been shown to be of poor quality. Nonetheless, countries such as Ecuador, Peru and Bolivia have all established governmental policies for Child Development (*Cuna Más* in Peru; *Crecer bien para vivir bien* in Bolivia; *Centros infantiles del Buen Vivir* in Ecuador), as well as developing programs to palliate any shortcomings as far as possible [3].

The *casas cuna* are a part of the public state care system in Bolivia aimed at helping the development of foster children aged between 0 and 5 years old who are living in vulnerable situations of psychological, social and family risk. In Bolivia, few studies have been published on the subject of the impact of institutional care and the care of children in these *casas cuna*. However, there has been research on the repercussions caused by teenage pregnancies, single parent families, violence, lack of stimulation and other psychosocial risks that can lead to poverty, unemployment and a low educational level among the parents [1,14,15,16,17,18,19].

The aim of this study was to evaluate the environmental quality of the *casas cuna* of the Department of Chuquisaca (Plurinational state of Bolivia) among children from 0 to 2 years of age after receiving an intervention program based on psychomotor therapy.

## 2. Materials and Methods

This study is a part of the 2016DEC018 research project funded by the Andalusian International Cooperation Agency of the Government of Andalucía (Spain) and has been approved by the Bioethics Committee of the University of Almería.

### 2.1. Sample

A quantitative approach was used, based on field observations of children in the *casas cuna* of the Department of Chuquisaca (Bolivia). These public centers foster underage children who are subject to various psychosocial risk situations, such as negligence, abuse, violence, being orphaned and similar. Ethical approval and authorization for this study was granted by the institutions that act as guardians and care providers of these children at risk. The inclusion criteria were applied to select children aged between 0 and 2 years old from a total population of 92 children aged between 0 and 6 years old who were fostered at two *casas cuna* within the department. The children were medically evaluated as being healthy (no illnesses affecting psychomotor development) and in a permanent internship situation at the homes due to abandonment, negligence or being orphaned. The exclusion criteria were children over the age of 2 years who suffered from any medical condition, chronic illness, established neurodevelopment disorder or whose stay was shorter than the study duration, thus preventing a systematic observation.

Once the previously cited criteria were applied, the final study sample comprised 36 children under the age of 2 years old, of whom 12 children were aged between 0 and 12 months of age (33% of the sample) and 24 were aged between 13 and 24 months (67% of the total sample). The characteristics of the sample are featured below (Table 1).

**Figure 1:**
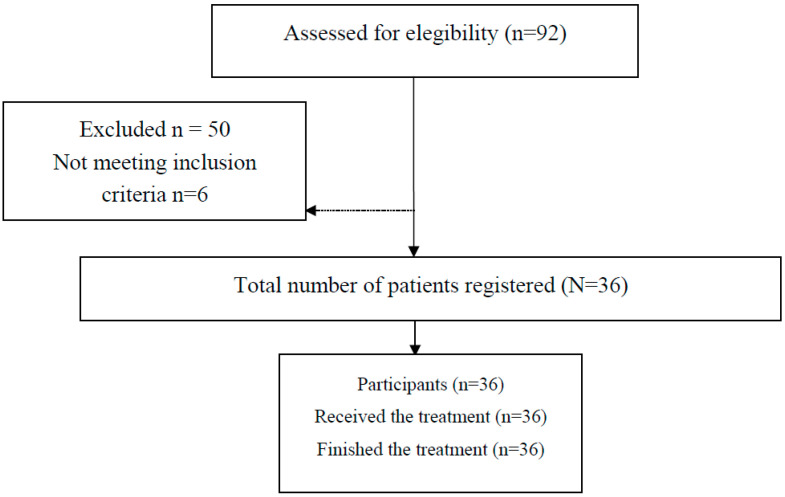
Study design flowchart.

The selection process is shown in Figure 1. All children came from low status socioeconomic environments.

Informed consent was obtained from the directors of both centers for the field research (according to the Declaration of Helsinki on ethical criteria for research).

### 2.2. Instruments

Data collection was based on the Infant/Toddler Home Observation for Measurement of the Environment (IT-HOME), developed by Caldwell and Bradley in 1984, translated and adapted to Spanish by Nieer [20]. This tool is highly useful in research performed in a Latin American context [5,20,21,22,23].

The IT HOME structure is based on six factors:

1. Responsivity (capacity of initiative): this refers to the extent of appropriate responsiveness of the parent to the child’s needs.
2. Acceptance: this refers to the degree of acceptance of suboptimal behavior.
3. Organization: this refers to the level of regularity and predictability of the environment.
4. Learning materials: this is based on whether appropriate games are provided that stimulate the infant’s development.
5. Involvement: defines the extent of adult involvement in learning and stimulation.
6. Variety: involves the inclusion of events and people who generate variety without disorganizing the child’s routine. All these indicators measure the environmental quality in which the child grows.

This scale measures the quality of the family context surrounding the child. It consists of 55 items divided into eight subscales. The items on this binary scale are scored with 0 (–) and 1 (+), denoting the absence or presence of the same. The information provided by the child’s mother or the adult in charge is entered to the recording sheet. In our case, the items were evaluated by direct observation by the interviewer.

Furthermore, the Attachment During Stress Scale (ADS) by Massie–Campbell was used, which is applied by government authorities for the prevention and care of psychosocial risk in Latin-American countries [4,24]. It is an observation guide that evaluates the appropriateness of the child–carer interaction from the age of six months and is based on a short period of observation time of between five and 15 min. The aim is to have clear observational and behavioral criteria, which are usually strong indicators for the quality of the affective bond or the attachment tendency between the mother/carer and the child.

The instrument contains six indicators:

1. Holding: the mutually reciprocated posturing of the infant and the mother while the infant is supported in the arms of the mother.
2. Gazing: refers to the exchange of gazes, their intensity and maintenance.
3. Vocalizing: refers to sounds, songs, babbling that are emitted when interacting, playing or communicating something.
4. Contact: skin to skin contact between the carer and the child.
5. Touching (a) the search for skin to skin contact between the mother and the child to interact, play, stimulate or calm. Touching (b) the avoidance of skin to skin contact that occurs between the carer and the child.
6. Proximity or closeness: this is the process of being close, far or next to one another, referring to the protector adult and the child.
7. Affect: facial expressions signaling emotional stress.

The scoring is from 1 to 5 points. Low scores are 1 or 2 in the case of a tendency to avoid attachment, scores of 3 or 4 indicate a tendency toward safe attachment and a score of 5 is a tendency toward anxious attachment [4].

### 2.3. Intervention

An initial individual assessment was performed with a duration of between 45 and 60 min per child during the months of May to June 2018. This assessment was supported by data collection and the assistance of staff specially trained in assessments, plus a team of six therapists from the neurodevelopmental project, who were external to the study.

The objective of applying psychomotor therapy was to influence the motor, cognitive, social and affective development of the child, support the relationship with the environment and consider the individual differences between the children, thus promoting the necessary emotional support to positively impact the subject [25].

The intervention program took place between July and November 2018. In total, 120 twice-weekly sessions were performed with a duration of 45 min each. These were applied to groups of three to four children by two therapists specifically trained in psychomotor therapy. The intervention was performed in a room specifically suited for the sessions within the *casas cuna* participating in the study.

The content of the sessions was based on a format of four key moments: an admission ceremony designed to decrease anxiety, to develop listening and waiting skills and to guide the session; followed by a second stage of sensorimotor activity in which early stimulation activities were performed according to the particular qualitative developmental need of each child, based on the use of sensory and cognitive circuits; a third therapeutic stage to enhance the learning experience, achieved by activities based on socio-affective stimulation based on contact, self-organization and permanence as precursors for a safe attachment. Finally, a closing ceremony, which marked the end to the activities and involved tidying up the materials and the farewell to the children from the therapeutic environment [26].

Upon completion of the intervention process, the HOME and ADS scales were applied once more to verify the results obtained post-intervention. The field work was performed via registers gathered via observation of the interactions between the child and the carer during the waking hours of the children and during periods of play, feeding, toileting and similar.

### 2.4. Statistical Analysis

The data were processed using the SPSS 21.0 (IBM, Armonk, New York, NY, U.S) statistical program, comparing the means obtained with the medians offered using the IT HOME as the reference and the average score of the ADS. Likewise, the statistical analysis was performed by a professional who was external to the study in order to preserve the impartiality of the results obtained.

## 3. Results

Table 2 features the results obtained on behalf of the sample evaluated using the IT-HOME scale with pre and post-intervention scores.

The Student’s *t*-test was applied, observing that t_(36)_ = −4.362; *p* < 001. The result was significant. An important increase was found in the mean of the items responsiveness, organization, learning materials and involvement. These significant differences equaled to a positive result regarding the development of interned children in the areas of their development, in that the total value of the scale showed a significant difference (positive change) regarding the situation prior to the intervention.

Table 3 displays the results obtained in the attachment scale. The results are shown prior and after the intervention in the dimensions referring to gaze, vocalization, manipulation (in two items: approximation to contact, avoidance of contact), supporting, affect and proximity.

For these results, the non-parametric Wilcoxon’s test was used, observing that the indicators for vocalization (*p* = 0.02), touching A (*p* = 0.025) and supporting (*p* = 0.014) showed lower values to the rest of the areas under study. An important increase was found in the tendency toward safe attachment in these three indicators, which was favorable for the child’s emotional development. In contrast, in the remaining items evaluated (given that these differences were not shown), it was not so evident that the type of attachment developed was appropriate for obtaining an appropriate neuro-psychosocial development, considering their age and social situation.

## 4. Discussion

Children who are institutionalized in *casas cuna* come from dysfunctional families, are orphans or have experienced intrafamily violence and, due to a variety of factors, are exposed to permanent stress, which affects their ability to build safe, effective and consistent emotional ties during everyday interactions. The explanation for this is based on studies on this subject [3,7] that report the effects of cortisol in children living in orphanages showing that without an appropriate stimulation of the external environment, the biological circuits become affected as well as the later development of intelligence and behavior [27,28].

Other authors studied the mental health of children from families with unfavorable socioeconomic levels or single parent families, finding a higher risk of exhibiting greater physical and mental health problems [27,29,30,31].

Culturally, in Bolivia, it is the mother who assumes the main role of raising the child during the first years of life, with a more permissive style in Guaraní culture and more authoritarian in the Aymara and Quechan cultures. In these two latter cases, abandonment of the child is socially disapproved of, together with the impossibility of pregnancy and childbirth, as children are thought to have an identity from conception and are considered an integral part of the community [3,9]. However, the development indicators are increasingly complex in the country, together with the field–city migration, where those in charge of raising children have a low educational level, which has effects on breastfeeding, supplementary feeding, language development, child’s play [13]. There are also the emotional effects of discipline to consider, which is often via physical punishment [3,8,9,13]. These data are quite similar to other studies performed in Latin American countries, such as Ecuador, Peru and Chile [16].

In the present study, it is shown that there is a lack of the necessary degree of response from the adult towards a child’s behavior which, in turn, negatively affects the appropriate overall development required in the first years of life. This reaffirms the direct relation between psychomotor development disorders (especially regarding motor skills, coordination, language and cognition) [7,11,27,28] with environmental factors that promote affective responses [13,16,32]. When the quality of the environment improves [1,3,17], this is positively reflected in socio-emotional aspects, such as vocalization, contact and sensory stimulation, which optimize the opportunities of a more balanced neurological, social, biological and emotional development [7].

The involvement that is generated on behalf of the carer in the interaction with the children under study should be based on the prevision of emotional and affective stability, the use of varied play materials, assertive responses during interactions and greater visual and affective contact, as direct threats to development can occur, generated by the absence of opportunities in the child’s immediate surroundings [27]. The environment offered by the *casas cuna*, which is the context of this study, does not provide the variety of activities for their developmental age. While the carers are present, they must display a greater affective involvement via attitudes of acceptance towards the children and a greater degree of response towards their daily needs, as well as the inclusion of a variety of activities at the center. A comparative analysis with the studies reviewed [7,16,17,27] confirms that environmental and family deprivation is positively related with moderate to severe developmental delays, while early stimulation favors the development of skills and competencies, which favors their comprehensive development [1,2,13,32].

In addition, the results shown by Faba [16] and confirmed by the macro study by Araujo [3] show that the quality of the environment in the child’s development during the first years of life go beyond the organic and that the relationship with the primary environment, such as the mother, is essential to harmonious development. In the present study, the children residents at the *casas cuna* are exposed to risk factors due to the scarcity of opportunities, absence of a safe family environment and the lack of stable stimuli in their first years of life. Moreno et al. [28] also studied the influence of the family environment on the motor development of infants aged 0 to 3 years who were accompanied by their mothers in prison, in which the abbreviated HOME scale was used, and found serious red flags regarding the negative influence that an inappropriate family environment had on the motor development of the child with regard to variables of responsiveness, acceptance, organization, learning materials, involvement and variety—all of which were examined in the present study and which primarily revealed a high incidence of negative responses. More specifically, all these directly influenced motor development as the results revealed that the environment directly impacts development and is deficient among the children fostered in *casas cuna* in Bolivia regarding variables of responsiveness, acceptance and variety.

In support of Bunkers [7], it is confirmed that children raised in biological families, foster families and adoptive families demonstrate better physical, intellectual development when compared to children living in institutional care. The results obtained in this study confirm the need to promote a better quality regarding the service, process and care provided by the staff in the *casas cuna* of Chuquisaca (Bolivia).

As noted by Miño [32] in 2016, it is obvious that neglect and emotional deprivation has an impact on the cognitive development of children in care homes. In this study, several similar variables were studied: institutionalization time, constant change of carers and fellow housemates and cultural and geographic factors, all of which affect the ability of children to establish appropriate bonds.

Thus, migration, poverty, the lack of resources and work opportunities and the low educational level of the parents constitute some of the variables that increase the psychosocial risk leading to a poor environment for the child and, therefore, a lack of appropriate stimulation [15]. It is important to reflect on the value of the environment as a mediator in the relationship between the quality of parental care and a safe affective bond, rather than only considering the influence of the genetic and/or biological factor. The need to act upon the socio-family risk is obvious, optimizing the context and the type of relational interactions that are generated from the same. Education and social policies must unify efforts in the optimization of early childhood development due to the undisputed importance of the intervention of contextual factors in the construction of healthy development in populations at psychosocial risk [11,32,33].

This study has several limitations. First, the constant changes in care and nursing staff, which varied due to work shifts; the unnecessary exposure of children with temporary volunteers who, far from offering variety, created instability in their interactions with the children; and, finally, the continuous arrival of new children coming to the *casas cuna* for short periods of time, which made it difficult to maintain a stable study sample during the research.

## 5. Conclusions

The quality of the environment in the *casas cuna* featured in this study was deficient for all the factors studied according to the IT HOME and demonstrated major deficiencies in terms of responsiveness, acceptance and variety of materials.

The factors relating mainly to objects, events and communicative interactions, which most directly impact the healthy development of a child, were not appropriate for their age. An under-stimulated environment prevails, which limits the possibilities of adaptation and overcoming their limitations and social status, experiencing the necessary social integration in order for the children to enjoy a healthy lifestyle. This lack of stimulation can lead to later learning difficulties, behavioral problems and insecure emotional attachments.

It is necessary to promote affective involvement according to the needs of children institutionalized in the *casas cuna* as they require a greater degree of response to their needs and a better preparation of the adults responsible for the care of the children. These measures protect and promote healthy interactions within the biopsychosocial model of comprehensive health and strengthen the prevention of socioenvironmental risk factors.

The timely and early intervention examined in this study offered favorable results, demonstrating that improvements in the quality of the environment favorably affected the children’s attachment. It is, therefore, essential for public infancy protection policies in social situations of vulnerability to consider children’s mental and socioemotional variables.

## Figures and Tables

**Table 1:** Displays the selection process performed to select the final study sample.

Characteristics	Participants
Sex	17 Boys 19 Girls
Height (cm)	Boys: 97 cm Girls: 91 cm Mean height: 93.8 cm
Weight (kilograms)	Boys: 7.0 kg Girls: 6.7 kg Mean weight: 6.5 kg
Age	0 to 12 months: 9 boys/11 girls Between 13 and 24 months: 8 boys/8 girls
Mean duration of institutionalization	From 0 to 12 months: 12 subjects (33%) From 13 to 24 months: 24 subjects (67%)

**Table 2:** Pre and post-intervention scores.

HOME	Measure, Mean (SD)		Mean Difference	Student’s *t*-Test		*d*
	Pre	Post		*t*_(36)_	*p*-Value	
Responsiveness	6.46 (1.91)	7.35 (1.6)	−0.89	−2.167	0.037	0.36
Acceptance	2.78 (1.93)	2.22 (1.9)	0.57	2.359	0.024	0.39
Organization	3.19 (0.84)	4.22 (0.71)	−1.03	−6.004	<0.001	0.99
Learning material	3.76 (4.07)	6.32 (3.33)	−2.57	−5.609	<0.001	0.92
Involvement	1.78 (1.75)	3.7 (1)	−1.92	−6.843	<0.001	1.12
Variety	1.51 (0.61)	1.7 (0.97)	−0.19	−1.022	0.314	0.17
Total	19.46 (10.19)	25.38 (6.29)	−5.92	−4.362	<0.001	0.72

**Table 3:** Results of the attachment indicators for the casas cuna.

ATTACHMENT	Measure, Mean (SD)		Wilcoxon’s Test	
	Pre	Post	*Z*	*p*-Value
Gaze	3 (3–5)	4 (3–5)	−0.08	0.937
Vocalization	3 (2–3)	3 (2–5)	−2.325	0.02
Touching A	2 (2–3)	3 (2–5)	−2.24	0.025
Touching B	5 (4–5)	5 (4–5)	−1.541	0.123
Supporting	3 (3–5)	4 (3–5)	−2.449	0.014
Affect	3 (3–4)	4 (4–4)	−1.694	0.09
Proximity	4 (2–5)	4 (2–4.5)	−0.544	0.586
